# Polyunsaturated Fatty Acids in Atrial Fibrillation: Looking for the Proper Candidates

**DOI:** 10.3389/fphys.2012.00370

**Published:** 2012-09-17

**Authors:** Oscar Salvador-Montañés, Alfonso Gómez-Gallanti, Daniel Garofalo, Sami F. Noujaim, Rafael Peinado, David Filgueiras-Rama

**Affiliations:** ^1^Cardiac Electrophysiology Unit, Department of Cardiology, Hospital Universitario la PazMadrid, Spain; ^2^Department of Internal Medicine, Center for Arrhythmia Research, University of MichiganAnn Arbor, MI, USA

**Keywords:** atrial fibrillation, omega-3 polyunsaturated fatty acids, remodeling, prevention, drug therapy

## Abstract

Atrial fibrillation (AF) is the most common sustained arrhythmia encountered in clinical practice with growing prevalence in developed countries. Several medical and interventional therapies, such as atrial specific drugs and pulmonary vein isolation, have demonstrated prevention of recurrences. However, their suboptimal long-term success and significant rate of secondary effects have led to intensive research in the last decade focused on novel alternative and supplemental therapies. One such candidate is polyunsaturated fatty acids (PUFAs). Because of their biological properties, safety, simplicity, and relatively cheap cost, there is a special clinical interest in omega-3 PUFAs as a possible antiarrhythmic agent. Obtained from diets rich in fish, they represent one of the current supplemental therapies. At the cellular level, an increasing body of evidence has shown that *n*-3 PUFAs exert a variety of effects on cardiac ion channels, membrane dynamic properties, inflammatory cascade, and other targets related to AF prevention. In this article, we review the current basic and clinical evidence pertinent to *n*-3 PUFAs in AF treatment and prevention. We also discuss controversial outcomes among clinical studies and propose specific subsets of AF patients who will benefit most from *n*-3 PUFAs.

## Introduction

Atrial Fibrillation (AF) is the most common sustained arrhythmia encountered in clinical practice (Kannel et al., 1998). In developed countries the arrhythmia is associated with doubling of mortality in both sexes and is one of the main causes of embolism (Wolf et al., 1991). Although AF already represents an important health care problem, its prevalence is estimated to increase in the next decade (Miyasaka et al., 2006). AF is classified by how long the patient has had the arrhythmia. AF that lasts less than 7 days is classified as paroxysmal. AF that lasts longer than 7 days, without a return to sinus rhythm, is designated as persistent. Persistent AF lasting for more than 1 year is termed long-standing AF as long as a rhythm control strategy is still pursued. However, when the rhythm control is no longer pursued the arrhythmia is designated as permanent AF (Camm et al., 2010). Current therapeutic options, either antiarrhythmic drugs (amiodarone, flecainide, vernakalant, dronedarone, etc.) or radiofrequency catheter-based procedures have limited efficacy and are not completely free of complications (Lafuente-Lafuente et al., 2006; Cappato et al., 2010). This is because as the arrhythmia perpetuates there is substantial atrial remodeling that promotes the maintenance of AF (Nattel et al., 2008).

Therefore, as the prevalence of AF increases, preventing its first episode or recurrences before it becomes permanent is a crucial component of treatment. Currently, therapies aimed at preventing recurrences (Healey et al., 2005; Lafuente-Lafuente et al., 2006; Singh et al., 2007) have not demonstrated an elimination of the arrhythmia burden or a wide safety profile. While in the last decade new multichannel profile and more atrial specific antiarrhythmic drugs have been developed, they are still not without substantial side effects and can be pro-arrhythmic (Kober et al., 2008). Therefore, novel therapeutic options are needed to improved AF management (Dobrev et al., 2012). An increasing amount of scientific evidence has suggested fish oil dietary supplement as a simple, safe, and relatively cheap adjunct to existing therapies (Mozaffarian et al., 2004; Calo et al., 2005; Nodari et al., 2011). Here, we aim to review the current knowledge regarding the role of polyunsaturated fatty acids (PUFAs) as a therapeutic used for the treatment and prevention of AF. Using a bench to the bedside approach, we will attempt to identify the appropriate AF candidates who will successfully respond to PUFAs.

## Biochemistry of Polyunsaturated Fatty Acids

Fatty acids are essential constituents of the cell membrane. Categorized by the length of their chains they are classified as short (fewer than 6 carbon atoms), medium (6–12 carbon atoms), or long (greater than 12 carbon atoms). They are further classified as saturated or unsaturated. Saturated fatty acids have no double bonds between their carbon atoms allowing the molecule to become “saturated” with the maximal number of possible hydrogen atoms attaching. Unsaturated forms of fatty acids possess biophysical, structural, and dynamic properties essential for normal cellular function (Leaf et al., 2005). For Omega-3 (*n*-3 fatty acids), the terminal double bond is on C3 counting from the methyl end of the hydrocarbon chain. The more commonly encountered *n*-6 (or omega-6) PUFAs have the terminal double bond on C6. The long hydrocarbon chain of the *n*-3/*n*-6 PUFAs, with multiple double bonds and with the first double bond occurring in the C3/C6 position result in complex and unique 3-dimensional configurations of PUFAs that are essential to their biological properties (Mozaffarian and Wu, 2011).

Fatty acids required for normal physiological functions that are produced by the body but obtained from food are called essential. Two of these are linoleic (*n*-6 PUFA) and α-linolenic (*n*-3 PUFA) acids. Once ingested, biochemical pathways can further metabolize essential fatty acids into long-chain more unsaturated derivatives. Linoleic acid can be converted into arachidonic acid and α-linolenic acid into eicosapentaenoic and docosahexaenoic acids (EPA and DHA, respectively; Burdge et al., 2002). However, endogenous conversion is very limited in humans (8% assuming the best scenario), which makes tissue and circulating EPA and DHA levels primarily dependent on direct dietary consumption (Burdge, 2004). While plants are a good source of linoleic and α-linolenic acids, longer chain *n*-3 fatty acids are mainly obtained from fish (tuna, herring, mackerel, etc.). In humans, fish oil supplements result in progressively higher proportions of EPA, DHA, and total *n*-3 PUFAs in atrial phospholipids, along with a reciprocal lowering of long-chain *n*-6 PUFAs, predominantly arachidonic acid (Metcalf et al., 2007). Animal and human studies demonstrate a progressive incorporation of *n*-3 PUFAs in the myocardial cell membrane over a 30-day period (Owen et al., 2004; Metcalf et al., 2007). Certain factors such as age, diabetes, body mass index, etc. could affect the incorporation of *n*-3 PUFAs into the cell membrane (Sands et al., 2005). Therefore, the slow and gradual incorporation of *n*-3 PUFAs into the membrane phospholipids should be taken into consideration when assessing the beneficial effects of PUFAs supplemental therapy, since a delay in protection can be expected. This has been observed when prevention of total mortality and sudden cardiac death was examined after the initiation of *n*-3 PUFA supplementation (Marchioli et al., 2002). Similarly, diverging results on the rate of recurrences after a DC shock in patients with persistent AF may also be partially explained by differences in the duration of *n*-3 PUFA therapy before cardioversion (Bianconi et al., 2011; Nodari et al., 2011).

## Cellular Effects of Polyunsaturated Fatty Acids

A large body of evidence supports the role of *n*-3 PUFAs in stabilizing the cardiomyocyte membrane and altering cardiac cells’ electrophysiology (Leaf et al., 2003). Interestingly, some experiments have demonstrated that *n*-3 PUFAs exert their effects without strong ionic or covalent binding to specific targets in the cell membrane. It is currently accepted that their incorporation within the hydrophobic tail of the cardiomyocyte membrane phospholipids is sufficient to elicit electrophysiological changes, and even antiarrhythmic action (Kang and Leaf, 1994). However, direct interaction with proteins of certain cardiac ion channels such as Nav1.5 has also been suggested by introducing single amino acid mutations in the wild type protein that significantly reduced the expected potency of EPA to inhibit the fast sodium current (*I*_Na_; Xiao et al., 2001).

The effects of PUFAs on cardiac ionic currents have been proposed as a major player in protection against AF. Acute effects of PUFAs on the biophysical properties of ion channels may substantially differ from those of chronically administered PUFAs that are gradually incorporated into the cell membrane (Den Ruijter et al., 2007). In human atrial cells, EPA and DHA acutely inhibit *I*_Na_ by shifting the potential of *I*_Na_ availability toward more negative voltages and increasing *I*_Na_ inactivation at resting states (Li et al., 2009). This effect on *I*_Na_ has also been observed in HEK 293 cells expressing human cardiac sodium channels and in neonatal rats (Xiao et al., 1995, 1998). In contrast with the acute effects, peak *I*_Na_ was unaffected by incorporated *n*-3 PUFAs in ventricular myocytes isolated from pigs and rats fed with a diet rich in fish oil (Leifert et al., 2000; Verkerk et al., 2006).

In addition to affecting *I*_Na_ the antiarrhythmic properties of PUFAs have also been attributed to their capability to modulate the L-type calcium channel (*I*_Ca-L_; Xiao et al., 1997) and the human ether-a-go-go-related gene (HERG) channel, which mediates the repolarizing rapid delayed rectifier K^+^ current (*I*_Kr_; Guizy et al., 2005). Similar to the effects described on *I*_Na_, no changes were observed on *I*_Kr_ density and calcium homeostasis (diastolic Ca^2+^ and Ca^2+^ transient amplitude) in pig ventricular myocytes with incorporated sarcolemmal *n*-3 PUFAs (Verkerk et al., 2006). In the same study and in line with the acute administration (Xiao et al., 2004), chronic administration of *n*-3 PUFAs resulted in reduced Na^+^-Ca^2+^ exchange current (*I*_NCX_). This reduction may explain the decreased propensity to develop delayed after depolarizations (Blaustein and Lederer, 1999). Moreover, changes in membrane phospholipids have been observed in rat atrial myocytes after a fish oil dietary supplement compared to saturated and monounsaturated diets. The fish oil dietary supplement was associated with calcium sparks of smaller area, and shorter duration in cells with a higher ratio of *n*-3 to *n*-6 PUFAs (Honen and Saint, 2002). This may contribute to prevent diastolic calcium release and decrease the propensity of delayed after depolarizations, which can initiate AF.

With regards to the other repolarizing potassium currents, in human atrial cells, EPA and DHA significantly inhibit the ultra-rapid delayed rectifier K^+^ current (*I*_kur_) and the transient outward K^+^ current (*I*_to_; Li et al., 2009). The inhibition of *I*_kur_ is of special interest since represents an atrial specific current (Li et al., 1996), and its blockade might lead to AF termination (Blaauw et al., 2004). Although attractive, specific *I*_kur_-blockade may not be effective to terminate persistent AF and certain AF models, in which *I*_Kur_ is decreased and increased inward rectifier K^+^ currents may counterbalance the potential effect of blocking *I*_Kur_ in action potential duration (Van Wagoner et al., 1997; Pandit et al., 2011). Chronic treatment with EPA at low concentrations (1 μM) may increase *I*_Kur_ current. Conversely higher concentrations of DHA and EPA (30 μM; more physiological) decrease the expression of Kv1.5 protein channel (principal molecular component of *I*_Kur_; Koshida et al., 2009).

In human atrial cells, no significant effects have been recorded in the main inward rectifier potassium current (*I*_K1_) after exposing the cells to oleic acid (Crumb et al., 1999). The same results were obtained in ferret cardiomyocytes after acute administration of *n*-3 PUFAs (Xiao et al., 2002). Conversely, incorporation of *n*-3 PUFAs into the sarcolemma results in *I*_K1_ increase by ≈50%, which shortens the action potential duration, reduces delayed after depolarizations and triggered activity (Verkerk et al., 2006; Den Ruijter et al., 2008). The slow delayed rectifier K^+^ current (*I*_Ks_) also shows significant increase after incorporation of *n*-3 PUFAs in the sarcolemma of ventricular myocytes (Verkerk et al., 2006). Acute effects on *I*_Ks_ differ depending on the type of *n*-3 PUFA. While DHA has shown to increase *I*_Ks_ magnitude, EPA does not exert any significant effect on *I*_Ks_ (Doolan et al., 2002).

Unsaturated free fatty acids such as oleic, linoleic, and arachidonic acids reversibly inhibited the ATP-dependent gating of native acetylcholine-sensitive K^+^ current (*I*_K-Ach_) in rat atrial cells (Kim and Pleumsamran, 2000). Free unsaturated fatty acids of the cardiac cell membrane seem to be crucial to keep the *I*_K-ACh_ channel in the short-lived, single open state, and maintain low channel activity despite the presence of ATP in the cell. The role of *I*_K,Ach_ in AF is well described in experimental models of AF. Activation of these channels causes hyperpolarization of the resting membrane potential and shortening of the action potential duration. Heterogeneous distribution of *I*_K,ACh_ channels in the atria generate non-uniform distribution of refractory periods and make the atria prompt to AF (Moe and Abildskov, 1959; Sarmast et al., 2003).

In addition, *n*-3 PUFAs might affect stretch activated channels (SAC), which are non-specific ion channels activated in response to mechanical deformation of the membrane. In rat atrial myocytes, membrane compliance increases after acute addition of the *n*-3 PUFAs DHA and EPA (Jahangiri et al., 2000). Langendorff-perfused hearts of rabbits on a dietary fish oil supplement were more resistant to stretch-induced AF compared to hearts from controls (Ninio et al., 2005). This is similar to the results of earlier experiments (Bode et al., 2001) after blocking SAC by the tarantula venom peptide GsMtx-4. Consequently, it could be speculated that an increase in membrane fluidity could protect against stretch-induced vulnerability to AF. However, it remains unknown whether PUFAs modify the biophysical properties of SAC.

Polyunsaturated fatty acids may also act through alternative mechanisms derived from their effects on inflammation, endothelial function, atherosclerosis, etc. Through the activation of transcription factors such as peroxisome proliferator-activated receptors (PPARs) and nuclear factor kappa B (NFκB), *n*-3 PUFAs are able to regulate metabolism and other cell and tissue responses, such as inflammation. Healthy human volunteers who undertook a dietary fish oil supplement showed decreased production of tumor necrosis factor-α (TNFα), interleukine-1β (IL-1β), IL-6, and various growth factors by monocytes or mononuclear cells that were stimulated with bacterial endotoxin (Endres et al., 1989; Trebble et al., 2003). Changes in cell membrane composition after *n*-3 PUFA supplementation alter the production and potency of eicosanoid and eicosanoid-like mediators produced from the *n*-6 PUFA arachidonic acid (prostaglandins, thromboxanes, and leukotrienes). These have well-established roles in the regulation of inflammation and immunity (Calder, 2006).

Therefore, based on acute effects and incorporation of *n*-3 PUFAs into the cardiac cell membrane, PUFAs may decrease atrial heterogeneity and Ca^+2^-induced triggered activity, both leading to lower risk of AF onset and recurrences. Altogether, cellular studies suggest that long-term *n*-3 PUFAs supplementation may act as an upstream therapy for substrate modification and membrane stabilization rather than a pure antiarrhythmic agent. Summarized acute and chronic *n*-3 PUFAs effects on cardiac ion currents are shown in Table 1.

**Table 1:** **Acute vs. chronic *n*-3 PUFAs effects or cardiac ion currents**.

Author/s Year	Ion Current	PUFAs Effects		Cell type
		Acute	Chronic	
Li et al. (2009); Xiao et al. (1995, 1998)	*I*_Na_	Decrease		Human atria, HEK 293, neonatal rat ventricular myocytes
Leifert et al. (2000); Verkerk et al. (2006)			Unaffected	Pig and rat ventricular myocytes
Li et al. (2009)	*I*_Kur_	Decrease		Human atria
Koshida et al. (2009)			Decrease (30 μM)	Transfected green monkey kidney fibroblast cells and rat atrium
Li et al. (2009)	*I*_to_	Decrease	Unknown	Human atria
Xiao et al. (1997)	*I*_Ca-L_	Decrease		Rat ventricular myocytes
Verkerk et al. (2006)			Decrease	Pig ventricular myocytes
Xiao et al. (2004)	*I*_NCX_	Decrease		HEK 293t
Verkerk et al. (2006)			Decrease	Pig ventricular myocytes
Guizy et al. (2005)	*I*_Kr_	Decrease		Chinese hamster ovary cells expressing HERG
Verkerk et al. (2006)			Unaffected	Pig ventricular myocytes
Doolan et al. (2002)	*I*_Ks_	Increase (DHA)		KvLQTl and hminK injected in Xenopus oocytes
Verkerk et al. (2006)			Increase	Pig ventricular myocytes
Xiao et al. (2002)	*I*_KI_	Unaffected		Adult ferret cardiomyocytes
Verkerk et al. (2006)			Increase	Pig ventricular myocytes
Kim and Pleumsamran (2000)	*I*_K-ACh_	Decrease	Unknown	Rat atrium
	SAC	Unknown	Unknown	

*SAC, stretch activated channels; DHA, docosahexaenoic acid*.

## Effects of Polyunsaturated Fatty Acids in Experimental Models of AF

Experimental models of AF show much less variability than human populations at large. This allows the identification of specific mechanisms or substrates suitable for potential treatment with PUFAs. Models mainly based on electrical remodeling, structural remodeling, or inflammatory-related models can provide valuable insights to understand the role of *n*-3 PUFAs in clinical AF.

Oral supplementation with *n*-3 PUFAs (DHA and EPA acids), commencing 2 weeks before tachypacing onset and continuing through the fast pacing period (7 days), did not significantly affect AF duration and atrial refractory period compared to sham-operated controls in a dog model of AF where the ventricular rate was controlled by atrioventricular block and ventricular demand pacing. Further, dogs that underwent ventricular tachypacing with the same regimen of *n*-3 PUFA supplementation showed decreased congestive heart failure-related atrial fibrosis and attenuated AF promotion induced by ventricular tachypacing (Sakabe et al., 2007). The authors found significantly decreased expression of phosphorylated mitogen-activated protein (MAP) kinases, which are particularly important in causing tissue fibrosis in both heart failure animals and AF patients (Goette et al., 2000; Petrich and Wang, 2004). In a dog model of AF with simultaneous fast atrial and ventricular pacing, *n*-3 PUFA supplementation resulted in less conduction time heterogeneity in the left atrium, and prevented pacing-induced increase in collagen turnover and collagen deposition in atrial appendages. PUFAs reduced both AF inducibility and duration of inducible AF (Laurent et al., 2008). Echocardiographic assessment of mechanical remodeling in those animals showed a similar decrease in left atrial-emptying function in treated animals and controls (Laurent et al., 2008).

However, there are discrepancies about the effects of PUFAs in the literature that appear to be model dependent. For example, acute administration of PUFAs prevented atrial electrical remodeling by significantly reducing the shortening of atrial effective refractory period caused by several hours of fast atrial pacing in dogs (Da Cunha et al., 2007). This acute effect was not observed in the same animal model of PUFAs treatment under long-term fast atrial pacing (Sakabe et al., 2007). Interestingly, both dietary supplements and acute administration of *n*-3 PUFAs prevented vagally induced AF in dogs (Sarrazin et al., 2007). Experimental results in vagally induced AF correlate with the relevant role of PUFAs in the cardiac cell membrane to modulate *I*_K,ACh_ as described above.

The antiarrhythmic effects of PUFAs have been observed in non-tachypaced models of AF as well. Accumulating evidence indicates that inflammatory pathways are of significance in AF. Although some evidence suggests that inflammation might be a causative agent for AF (Sata et al., 2004), a substantial body of evidence supports that AF and inflammatory pathways have a bidirectional relationship (Friedrichs et al., 2011). After cardiac surgery, leukocytosis and pro-inflammatory cytokines have been directly related to the incidence of post-operative AF. As the cytokines raise, the risk of post-operative AF concomitantly increases (Ishida et al., 2006). *n*-3 PUFAs show anti-inflammatory effects that may prevent AF episodes related to a highly inflammatory environment. Experimentally, in a canine model of open-chest sterile pericarditis, oral PUFAs supplement for 4 weeks before the operation and 2 days afterward resulted in less AF inducibility and maintenance than in a control group under regular feeding. Before the operation, there were no significant differences in conduction time, atrial effective refractory period (AERP; defined as the longest S_1_–S_2_ coupling interval that fails to depolarize the atria) and inflammatory markers between PUFAs group and controls. Two days after surgery, C-reactive protein (CRP), IL-6, and TNF-α levels were significantly lower in the PUFAs group. PUFAs supplementation also resulted in longer AERP and shorter intra-atrial conduction time after surgery (Zhang et al., 2011).

## Mechanisms Underlying AF and Their Link to Cellular and Experimental PUFAs Effects

Although the mechanisms underlying AF are not completely understood, the arrhythmia is believed to be reentrant. There is increasing evidence supporting the role of a unique or small number of functional reentrant sources (rotors) maintaining the arrhythmia (David Filgueiras Rama and José Jalife, 2011). This is largely because of the elucidation of the molecular mechanisms of reentry. Theoretically, it has been known that shortening of the action potential duration and increasing excitability can facilitate reentry (Pandit et al., 2005). However, it was not until the last decade that the role of inward rectifier K^+^ currents, such as *I*_K,ACh_ or *I*_K1_, and their ability to increase reentrant frequency and facilitate AF became a well-established molecular mechanism responsible for AF. For a complete review on the role of inward rectifiers (see Ehrlich, 2008; Jalife, 2011). While *I*_K,ACh_ may have a preferential role in paroxysmal AF and explain left-to-right differences in rates of activation (Voigt et al., 2010), the current seems to decrease in persistent AF. However, ionic remodeling leads to an increase in *I*_K1_ and constitutive active *I*_K,ACh_ (Dobrev et al., 2001, 2005; Makary et al., 2011). Conversely, as AF becomes persistent extensive data show decrease in *I*_to_, *I*_Kur_, *I*_Na_, and the L-type Ca^+2^ current (Van Wagoner et al., 1997, 1999; Dobrev et al., 2001; Sossalla et al., 2010).

While reentry seems to perpetuate the arrhythmia, Ca^2+^-dependent triggered activity may initiate AF. While the spontaneous release of Ca^+2^ and triggered activity implicate abnormal sarcoplasmic reticulum (SR) Ca^+2^ release as a trigger, the frequency of triggered activity, and spontaneous Ca^+2^ release are much slower than the typical AF activation rate (<1 vs. 6–9 Hz, respectively; Atienza et al., 2006, 2009; Voigt et al., 2012). As a result, it is unlikely they are the mechanism maintaining the arrhythmia. This idea is further supported by AF models like stretch-induced AF, in which a more depolarized resting membrane potential and the activation of SAC enable the generation of triggered activity. Even in the presence of a high rate of focal activity reentry was required to sustain AF (Filgueiras-Rama et al., 2012).

However, it is important to note that as the arrhythmia persists, electrical remodeling and functional changes in subcellular structures lead to higher susceptibility to Ca^2+^-dependent triggered activity. Ryanodine (RyR2) dysfunction and SR Ca^2+^ leak may contribute to further paroxysms and persistence of AF. Under certain conditions of excitability, anatomic and functional obstacles may interfere with propagation of regular or Ca^2+^ dependent waves, which may cause the formation of self-sustained vortices. Concomitantly, larger inward Na^+^-Ca^2+^-exchange current (*I*_NCX_) for a given SR Ca^2+^ release further increase the likelihood of delayed after depolarizations and triggered activity (Voigt et al., 2012).

In addition to electrical remodeling, structural changes can facilitate the long-term maintenance of AF (Nattel et al., 2008). Structural changes include atrial dilatation and an increase in atrial fibrosis. This is a consistent finding in AF models associated with congestive heart failure (Morillo et al., 1995; Li et al., 1999). Extracellular matrix dysregulation and atrial fibrosis increases atrial conduction heterogeneity and plays an important role in stabilizing reentry and making larger areas of the atria suitable for harboring reentry. Different inter related signaling pathways appear to be involved in the development of atrial fibrosis. The most prominent pathways studied are the renin-angiotensin system (RAS), transforming growth factor-β1 (TGF-β1), and the inflammation/oxidative stress pathways (Lin and Pan, 2008). Furthermore, elevated inflammatory mediators varied according to the different sub-types of the arrhythmia. There is a graded increase in N-terminal pro-brain natriuretic peptide (NTpBNP) and TNF-α with the duration and type of AF (permanent > persistent > paroxysmal). Patients with lone AF (no overt structural heart disease) are less likely to have elevated concentrations of biomarkers (IL-10, TNF-α, and NTpBNP; Li et al., 2010). Baseline levels of biomarkers and further decrease after cardioversion may also be used to predict sinus rhythm maintenance during the following year in patients with lone AF (Leftheriotis et al., 2009). However, structural remodeling does not occur in all AF models. Predominant electrical remodeling with minimal or no changes in atrial fibrosis are present in tachypacing induced AF models even after long periods of fast pacing, as long as tachycardiomyopathy is not present (Ausma et al., 1997).

Synthesizing all this together, the role of *n*-3 PUFAs in terminating AF by modulation of cardiac ion channels seems unlikely based on the limited effects observed with chronic PUFAs supplements. Moreover, once AF is initiated, the increase in *I*_K1_ after PUFAs supplement may facilitate reentry (Verkerk et al., 2006). However, *n*-3 PUFAs have also been shown to prevent AF, and it is speculated that an increase in membrane fluidity and *I*_K1_ may protect against stretch-induced triggered activity and AF initiation. Similarly, a decrease in abnormal Ca^+2^ release may also prevent focal triggered activity initiating AF (Honen and Saint, 2002). The crucial role of PUFAs to control ATP-induced increase in K_ACh_ channel activity may also be important in persistent AF, in which *I*_K,ACh_ is constitutively active. Additionally, it has been proposed that the PUFAs antiarrhythmic effects lie in the ability of *n*-3 PUFAs to modify the atrial substrate that perpetuates the arrhythmia. Thus, PUFAs have the capability to attenuate the inflammatory cascade and adverse remodeling occurring in response to mechanical stress (Sakabe et al., 2007; Laurent et al., 2008).

## Clinical Implications of PUFAs in AF Population

The AF population represents a large source of variability. Therefore, specific AF subsets could benefit more than others from using *n*-3 PUFA dietary supplementation. It is not surprising there are different responses to PUFAs depending on the type of AF (lone, paroxysmal, persistent, and permanent). Further, the evaluation of the efficacy of PUFAs is convoluted by concomitant therapies, degree of inflammatory biomarkers, and time-course of the supplementary dietary therapy before outcomes analysis. Based on the experimental studies we have discussed so far, we speculate that PUFAs are not predominantly antiarrhythmic ion channel blockers. Rather, they play an important role to prevent AF onset in disease states with strong inflammatory and structural remodeling components. Results from different trials highlight the potential role of *n*-3 PUFAs in preventing AF onset and recurrences mainly from persistent AF populations, which supports their role as upstream therapy.

The study by Calo et al. (2005) in patients who underwent coronary artery bypass graft (CABG) surgery showed that *n*-3 PUFA supplementation (EPA/DHA ratio 1:2) at least 5 days before surgery reduced the incidence of post-operative AF by ≈50%. Further, it was associated with a shorter hospital stay (Calo et al., 2005). Similar results were observed in a randomized trial with the same type of surgery and same PUFAs regimen (Sorice et al., 2011). Conversely, another randomized clinical trial in patients who underwent CABG surgery did not show statistical differences between *n*-3 PUFAs and placebo groups (Saravanan et al., 2010). This discrepancy may be explained by different designs of the studies. The EPA/DHA supplementation ratio was 1:2 in Calò’s study and 1.2:1 in the study by Saravanan et al. (2010). Considering that DHA may have greater impact on AF prevention (Virtanen et al., 2009), the results may be somehow affected by the study design. It also should be noted that Calò’s study included AF episodes lasting >5 min compared to >30 s in Saravanan’s. This resulted in a much higher AF incidence, without a clear clinical significance of such short episodes. Finally, the use of concomitant β-blockers and statins was much lower in the study by Calò (85 and 98% compared with 57 and 56%). Optimization of these agents could have decreased the beneficial effects of *n*-3 PUFAs.

Two other randomized trials in patients who underwent cardiac surgery either CABG and/or valvular replacement did not show any beneficial effect of *n*-3 PUFAs (EPA/DHA ratio 1.2/1.4:1) in preventing post-operative AF compared to controls (Heidarsdottir et al., 2010; Farquharson et al., 2011). The study by Heidarsdottir et al. (2010) was carried out in an Icelandic population with approximately 80% of participants taking cod liver oil or other fish oils, which resulted in very small changes of plasma *n*-3 PUFAs concentrations after the regimen. In the second study by Farquharson et al. (2011) the results showed a trend to toward a decrease in incidence of AF in the *n*-3 PUFAs group. However the sample size was underestimated for the AF rates observed in the study, which resulted in no statistically significant differences. The discussed clinical studies evaluating new-onset AF following cardiac surgery are summarized in Table 2.

**Table 2:** **Clinical trials in new-onset AF following cardiac surgery**.

Author year	Age (years)	Design	Study population	PUFAs dosage	No of patients	Duration	Results
Calo et al. (2005)	65.6 ± 8.5	Open label/randomized	Pre/post-CABG, SR	EPA/DHA 1:2	160	At least 5 days before CBAG until discharge	Reduced post-CABG surgical AF and shorter hospitalization
Saravanan et al. (2010)	66 (58–73)	Double blind/randomized	Pre/post-CABG, SR	EPA/DHA 1.2:1	108	At least 5 days before CBAG until discharge/5 days	No reduction in AF after CABG surgery
Heidarsdottir et al. (2010)	67 (43–82)	Double blind/randomized	Pre/post-cardiac surgery, SR	EPA/DHA 1.2:1	168	5–7 days before CABG and/or valvular repair until discharge/14 days	No reduction in AF after cardiac surgery
Farquharson et al. (2011)	64 ± 11	Double blind/randomized	Pre/post-cardiac surgery, SR	EPA/DHA 1.4:1	194	3 weeks before CABG and/or valve replacement until discharge/6 days	Trend to decrease in post-surgical AF. Decreased length of stay in the lCU
Sorice et al. (2011)	63 ± 10	Open label/randomized	Pre/post-cardiac surgery, SR	EPA/DHA 1:2	201	At least 5 days before CBAG until discharge	Decrease in “on-pump” CABG surgical AF

*AF, atrial fibrillation; CABG, coronary artery bypass graft surgery; DHA, docosahexaenoic acid; EPA, eicosapentaenoic acid; ICU, intensive care unit; PUFA, omega-3 polyunsaturated fatty acids; SR, sinus rhythm*.

Beyond post-operative AF, over a 12-year follow-up period regular consumption of *n*-3 PUFAs (tuna and other broiled or baked fish ≥5 times per week) showed ≈30% lower incidence of AF among subjects older than 65 years after adjustment for other risk factors (Mozaffarian et al., 2004). Although those observational results were encouraging, subsequent randomized trials brought up controversial results. The study by Nodari et al. (2011), in patients with persistent AF and at least one relapse after cardioversion, showed significant decrease in AF recurrences after cardioversion and *n*-3 PUFA supplementation (EPA/DHA ratio 1.2:2). More recently, in a similar AF population, the study by Kumar et al. (2012) showed that fish oil supplementation resulted in a sixfold prolongation in the median time to AF recurrence compared to controls. Conversely, two other randomized trials by Kowey et al. (2010); Bianconi et al. (2011) did not show any beneficial effect of *n*-3 PUFAs in preventing AF recurrences in persistent AF patients after cardioversion or in sinus rhythm patients with previously documented AF, respectively. Again, several methodological factors may partly explain these discordant findings. Nodari’s and Kumar’s studies included patients taking *n*-3 PUFA supplementation for at least 4 weeks before cardioversion, with a history of at least one previous cardioversion. Nodari’s series also enrolled a population with high prevalence (≈90%) of structural heart disease. Most patients in Bianconi’s study (≈60%) were experiencing their first episode of AF and only 25% of patients had a previous cardioversion. In addition, before cardioversion, the length of *n*-3 PUFAs therapy was shorter than the time required for incorporation of PUFAs in the cell membrane (≈28 days). This may explain why the majority of recurrences occurred very early in follow-up (2–3 weeks), before the expected biological effects of *n*-3 PUFAs. In addition, in both studies with negative results the presence of structural heart disease was significantly lower than in Nodari’s study, which may represent a key factor in understanding the potential role of PUFAs in preventing structural changes preferentially in patients with significant structural heart disease, similarly to the additional clinical benefits of spironolactone therapy in patients with AF and structural heart disease (Williams et al., 2011). The clinical studies evaluating AF incidence and recurrences are summarized in Table 3.

**Table 3:** **Main clinical trials in non-postoperative AF onset and recurrent AF**.

Author year	Age (years)	Design	Study population	PUFAs Dosage	No of patients	Duration	Results
Mozaffarian et al. (2004)	72(65–100)	Observational prospective	Population-based	Tuna, baked fish	4815	12 years	Lower incidence of AF with fish intake ≥1 time per week
Virtanen et al. (2009)	52.8 ± 5.3	Observational prospective	Population-based men. SR	Fish intake	2174	17.7 years	High serum levels of *n*-3 PUFAs decrease hospital diagnosis of AF
Kowey et al. (2010)	60.5 ± 12.8	Double blind/Randomized	Symptomatic paroxysmal/persistent AF, SR	EPA/DHA 1.2:1	663	24 weeks after enrollment	No reduction in recurrent AF
Nodari et al. (2011)	69.5 ± 7	Double blind/Randomized	Recurrent persistent AF >1 month + ACE-Is + Amiodarone	EPA/DHA 1.2:1	199	1 year	Decrease in persistent AF recurrences post-cardioversion
Bianconi et al. (2011)	69.2 ± 7.9	Double blind/Randomized	Persistent AF >1 month. Mainly “lone AF”	EPA/DHA 1.2:1	204	6 months after enrollment	No reduction in persistent AF recurrences post-cardioversion
Kumar et al. (2012)	61 ± 13	Open label/Randomized	Persistent AF >1 month ± ACE-Is/Amiodarone/Sotalol	EPA/DHA1.3:1	178	1 year	Decrease in persistent AF recurrences post-cardioversion

*ACE-Is, angiotensin-converting enzyme inhibitors; AF, atrial fibrillation; DHA, docosahexaenoic acid; EPA, eicosapentaenoic acid; PUFA, omega-3 polyunsaturated fatty acids; SR, sinus rhythm*.

## Future Directions

Data from animal studies seem to support the role of *n*-3 PUFAs as a therapeutic option in AF. AF models with intense structural remodeling due to heart failure and ventricular tachypacing, post open-chest surgery, and vagally induced AF have shown to benefit from *n*-3 PUFA supplementation. These effects are also supported by some of the effects of *n*-3 PUFAs at the cellular level. However, more mechanistic insights are necessary to further understand the specific pathways that make *n*-3 PUFAs an effective adjunctive therapy to prevent AF. Arguably, this is because structural remodeling of the atria and acute inflammation may affect AF susceptibility in a different manner based on the inflammatory pathways involved. In fact, recent data have shown that some of those inflammatory markers affect the activity of certain cardiac ion channels, as well as the interaction between cardiomyocytes and fibroblasts (Ottaviano and Yee, 2011; Ramos-Mondragon et al., 2011). As a result new experimental data is needed to understand how *n*-3 PUFAs influence the release of inflammatory markers and how those markers influence AF.

Changes in lipid composition of the sarcolemma seem to modulate some cardiac ion currents such as *I*_K,ACh_ and at least indirectly affect membrane susceptibility to stretch (Kim and Pleumsamran, 2000; Ninio et al., 2005). Specific changes in *n*-3 PUFAs composition in the lipid membrane of cardiomyocytes after dietary supplementation are currently unknown and need to be addressed. It is also necessary to study the specific *n*-3 PUFAs effects on SAC beyond the increase in membrane compliance.

Finally, DHA and EPA seem to exert different effects at the cellular level and clinical outcomes (Virtanen et al., 2009). New clinical trials based on mechanistic effects described in experimental studies will be necessary to avoid confounding factors that appear to be present in the current clinical trials.

## Conclusion

Biochemical and biophysical properties of *n*-3 PUFAs give rise to a variety of effects at the cellular and organ levels. They include increasing cardiomyocyte membrane stability, modulation of the biophysical properties of ion channels/cellular substructures, and significant effects on the inflammatory/fibrosis signaling pathways. In light of experimental and clinical studies, the latter may be especially important in preventing AF (post-operative or clinical recurrences). Consequently, dietary supplementation of *n*-3 PUFAs may be considerably more beneficial in patients with pronounced structural heart disease and atrial remodeling, high levels of inflammatory biomarkers, and low baseline levels of circulating PUFAs. New ongoing clinical trials, mainly focus on AF recurrences and post-operative AF, will hopefully help to pinpoint the specific subset of AF population that will benefit most from *n*-3 PUFA supplementation.

## Conflict of Interest Statement

The authors declare that the research was conducted in the absence of any commercial or financial relationships that could be construed as a potential conflict of interest.
